# The utility of the Rapid Emergency Medicine Score (REMS) compared with three other early warning scores in predicting in-hospital mortality among COVID-19 patients in the emergency department: a multicenter validation study

**DOI:** 10.1186/s12873-023-00814-w

**Published:** 2023-04-26

**Authors:** Onlak Ruangsomboon, Nutthida Phanprasert, Supawich Jirathanavichai, Chanokporn Puchongmart, Phetsinee Boonmee, Netiporn Thirawattanasoot, Thawonrat Dorongthom, Nattakarn praphruetkit, Apichaya Monsomboon

**Affiliations:** 1 Department of Emergency Medicine, Faculty of Medicine, Siriraj Hospital, Faculty of Medicine, Siriraj Hospital, Mahidol University, Mahidol University, Bangkok, Thailand; 2 Department of Emergency Medicine, Banphaeo General Hospital, Samutsakhon, Thailand; 3 Department of Emergency Medicine, Ratchaburi Hospital, Ratchaburi, Thailand; 4 Department of Emergency and Forensic Medicine, Buddhachinaraj Hospital, Phitsanulok, Thailand; 5 Department of Emergency Medicine and Forensic Medicine, Prachuap Khiri Khan hospital, Prachuap Khiri Khan, Thailand

**Keywords:** covid-19, Early warning score, Rapid emergency medicine score

## Abstract

**Background:**

Many early warning scores (EWSs) have been validated to prognosticate adverse outcomes of COVID-19 in the Emergency Department (ED), including the quick Sequential Organ Failure Assessment (qSOFA), the Modified Early Warning Score (MEWS), and the National Early Warning Score (NEWS). However, the Rapid Emergency Medicine Score (REMS) has not been widely validated for this purpose. We aimed to assess and compare the prognostic utility of REMS with that of qSOFA, MEWS, and NEWS for predicting mortality in emergency COVID-19 patients.

**Methods:**

We conducted a multi-center retrospective study at five EDs of various levels of care in Thailand. Adult patients visiting the ED who tested positive for COVID-19 prior to ED arrival or within the index hospital visit between January and December 2021 were included. Their EWSs at ED arrival were calculated and analysed. The primary outcome was all-cause in-hospital mortality. The secondary outcome was mechanical ventilation.

**Results:**

A total of 978 patients were included in the study; 254 (26%) died at hospital discharge, and 155 (15.8%) were intubated. REMS yielded the highest discrimination capacity for in-hospital mortality (the area under the receiver operator characteristics curves (AUROC) 0.771 (95% confidence interval (CI) 0.738, 0.804)), which was significantly higher than qSOFA (AUROC 0.620 (95%CI 0.589, 0.651); *p* < 0.001), MEWS (AUROC 0.657 (95%CI 0.619, 0.694); *p* < 0.001), and NEWS (AUROC 0.732 (95%CI 0.697, 0.767); *p* = 0.037). REMS was also the best EWS in terms of calibration, overall model performance, and balanced diagnostic accuracy indices at its optimal cutoff. REMS also performed better than other EWSs for mechanical ventilation.

**Conclusion:**

REMS was the early warning score with the highest prognostic utility as it outperformed qSOFA, MEWS, and NEWS in predicting in-hospital mortality in COVID-19 patients in the ED.

**Supplementary Information:**

The online version contains supplementary material available at 10.1186/s12873-023-00814-w.

## Introduction

The Coronavirus disease 2019 (COVID-19), an infectious disease caused by the severe acute respiratory syndrome coronavirus 2 (SARS-CoV-2), has spread globally infecting millions of people [1]. Its outbreak is considered a public health emergency of international concern that has caused multifaceted damages to healthcare systems worldwide [2]. The Emergency Department (ED) worldwide has faced unprecedented circumstances in which its healthcare personnel have to provide care for a significant number of patients overloading the capacity of their healthcare facilities, as demonstrated by, for example, generally longer lengths of stay in Australian ED and more overcrowded days in French ED [3, 4]. Early recognition of patients with greater severity who are at risk of developing adverse outcomes will not only improve the management and patients’ outcome but also facilitate patient flow, thereby decreasing ED overcrowding.

Early warning scores (EWSs) consisting of multiple physiologic variables readily available in clinical care are designed to help diagnose diseases and/or prognosticate their outcomes early in the disease progression. Many were developed and validated for use in the prehospital and emergency settings, such as the Systemic Inflammatory Response Syndrome (SIRS) criteria and the quick Sequential Organ Failure Assessment (qSOFA) scores, which are EWSs for diagnosing sepsis [5–7]. Some others, for example, the National Early Warning Score (NEWS) and the Modified Early Warning Score (MEWS), were developed to assess and monitor hospitalized patients for early detection of clinical deterioration [8, 9]. Nonetheless, these EWSs have also been validated to risk stratify patients with multiple conditions in the ED.[10, 11]. While the Rapid Emergency Medicine Score (REMS), an EWS developed to predict in-hospital mortality in non-surgical ED patients, [12] has also been validated and proved to be comparable, or even superior, to other EWSs in predicting adverse outcomes due to sepsis in the ED [13].

With the COVID-19 pandemic, many of these EWSs have been studied as means of aiding triage decision-making for COVID patients [14–19]. NEWS and its derivatives have been widely validated and recommended for triage decisions in patients with COVID-19 in some guidelines as it can accurately predict adverse outcomes during hospital admission, such as intensive care unit (ICU) admission, mechanical ventilation, and mortality [20, 21]. However, limited studies have explored the utility of REMS as an EWS prognosticating outcomes for COVID-19 patients. We hypothesized that REMS, with an inclusion of age as a component, should have even higher, or at least similar, prognostic utility for COVID-19 compared with NEWS, its derivatives, and other EWSs. Although some previous studies have shown that REMS could yield similar prognostic utility for COVID-19 compared to the other EWSs, most of those studies were single-centered with small sample sizes or were not conducted in the ED setting [22–25]. Therefore, we aimed to validate and compare the clinical utility of REMS, NEWS, MEWS, and qSOFA in predicting in-hospital mortality and mechanical ventilation in ED patients with COVID-19 infection.

## Methods and analysis

### Study design and setting

The study was a multicenter retrospective observational study conducted at five EDs in Thailand between January 2, 2021, and December 31, 2021. Participants were enrolled from a wide range of EDs, including those of university hospitals and other secondary and tertiary centers across different geographical areas in Thailand. Participating centers were the EDs of (1) Siriraj Hospital, Mahidol University, the largest tertiary university hospital in Thailand located in Bangkok, (2) Banphaeo Hospital, a large general hospital in Samutsakhon province, (3) Ratchaburi Hospital, the main provincial and teaching hospital in Ratchaburi province, (4) Buddhachinaraj hospital, a tertiary regional advanced-level and teaching hospital in Phitsanulok province, and (5) Prachuap Khiri Khan hospital, a general standard-level hospital of Prachuab Khiri Khan province. All participating centers provided healthcare services for both patients visiting from their residences and those transported by emergency medical services or referred from other lower-acuity centers. The study was approved by the Central Research Ethics Committee (CREC) of Thailand (certificate number CREC044/2022). Inform consent was waived as per the retrospective nature of the study. This cohort has previously been investigated for factors associated with poor outcomes comparing between elderly and non-elderly patients, and that study has been published [26].

### Participants

All adult patients (18 years of age and above) presenting to the ED with COVID-19 infection diagnosed with real-time polymerase chain reaction (RT-PCR) within the index ED visit or during hospital admission of the index ED visit were consecutively included. Those with unknown or unconfirmed COVID-19 status during the hospital stay of the index ED visit were excluded.

### Recruitment procedure and data collection

Consecutive patients who visited the participating EDs and had COVID-19 infection were included in the study. The duration of recruitment began from the visit date that the first patient diagnosed with COVID-19 during the largest waves of the pandemic in Thailand in the calendar year 2021. The study investigators at each participating center retrospectively reviewed consecutive ED patients’ records to identify eligible patients during the study period. Participants were identified by using ICD codes and by searching through the COVID-19 patient registry of each hospital.

Patients’ baseline demographics and physiologic variables collected upon their ED visits consisting of components of all EWSs, and the study outcomes were recorded using a standardized electronic case record form. Electronically recorded data were double-checked by another study coordinator for quality control.

### Scoring systems

qSOFA is a 3-item score consisting of respiratory rate (RR), mental status and systolic blood pressure (SBP); each item contains 1 point (0–3 points). NEWS, MEWS, and REMS are scoring systems with multiple components that use weighted score points for each component. NEWS (0–20 points) comprises of pulse rate (PR), RR, body temperature (BT), SBP, oxygen saturation and need for oxygen supplement. MEWS (0–14 points) has similar components to NEWS, namely RR, PR, BT, and SBP, but also with mental status. REMS consists of PR, RR, mean arterial pressure, mental status, pulse oximetry, and age (0–26 points). Table S1 elaborates the components and details of all the risk scores included in this study.

### Outcome measures

The primary outcome was mortality within the index hospital visit. The secondary outcome was the need for mechanical ventilation.

### Statistical analyses

Descriptive statistics were employed to describe the patients’ characteristics compared between patients discharged dead and alive. Categorical data are reported as frequency and percentage. Continuous variables are reported as mean and standard deviation (SD) or median and interquartile range, as appropriate. Between-group comparisons were performed using the Chi-squared or Fisher’s Exact test for categorical data and an independent t-test or the Mann-Whitney U test for continuous data.

We assessed the predictive performance of qSOFA, NEWS, MEWS, and REMS for primary and second outcomes. Discrimination is reported with area under the curve of the receiver operator characteristics curves (AUROC), from which we estimated the 95% confidence interval (CI) and made comparisons between EWSs. We also evaluated calibration with calibration plots and the Hosmer-Lemeshow test. Moreover, Nagelkerke’s R squared was used to evaluate overall model performance.

We assessed the clinical usefulness at the optimal cutoff values for all EWSs by reporting sensitivity, specificity, positive likelihood ratio (LR+), negative likelihood ratio (LR-), negative predictive value (NPV) and positive predictive value (PPV). These values were calculated for all EWSs at the cut-point according to the optimal Youden index. Pre-specified subgroup analyses were performed by excluding patients with do-not-resuscitate (DNR) status and by study center.

We performed all statistical analyses using SPSS version 18.0 (Chicago, IL., USA), R software version 3.6.1 (R Foundation for Statistical Computing, Vienna, Austria) with the rms, Hmisc, foreign, pROC, sciplot, and dca packages, and MedCalc for Windows version 19 (MedCalc statistical software, Mariakerke, Belgium).

## Results

### Study population

A total of 978 COVID-19 patients visited the participating EDs between January 2, 2021, and December 31, 2021, and were included in the study. There were no patients with missing study outcomes. Of all included patients, 254 (26.0%) met the primary outcome of all-cause in-hospital mortality, and 155 (15.8%) required mechanical ventilation. Patient characteristics are reported in Table 1. Patients who had mortality were older and had a higher prevalence of most underlying diseases. They also had significantly more severe abnormal initial vital signs, as well as a higher ICU admission rate compared to patients discharged alive.

**Table 1:** Baseline characteristics of emergency patients with COVID-19

Characteristic	Dead (254)	Alive (724)	*p*-value
Age	72.9 ± 15.1	58.6 ± 17.6	< 0.001
Sex (male)	149 (58.7)	343 (47.4)	0.002
Body mass index (kg/m^2^)	25.1 ± 6.7	26.1 ± 6.1	0.032
Day of symptoms	3, 3.25	4, 5	0.001
Underlying disease			
Diabetes mellitus	86 (33.9)	237 (32.7)	0.743
Coronary artery disease	34 (13.4)	56 (7.7)	0.007
Cerebrovascular disease	40 (15.7)	52 (7.2)	< 0.001
CKD stage 3–5 or ESRD	63 (24.8)	82 (11.3)	< 0.001
Chronic lung disease	29 (11.4)	52 (7.2)	0.035
Malignancy	23 (9.1)	41 (5.7)	0.06
Immunodeficiency	9 (3.5)	21 (2.9)	0.609
Do-not-resuscitate status	163 (64.2)	65 (9.0)	< 0.001
Vital signs and mental status at ED arrival			
Body temperature (^o^C)	37, 1.2	36, 1	0.02
Respiratory rate (breaths/min)	31.9 ± 7.8	29.2 ± 7.8	< 0.001
Pulse rate (beats/min)	98.9 ± 21.4	94.6 ± 19.0	0.002
Systolic blood pressure (mmHg)	136.6 ± 30.2	136.5 ± 28.5	0.942
Diastolic blood pressure (mmHg)	75.9 ± 18.8	79.6 ± 15.7	0.002
Oxygen saturation (%)	88, 17	94, 7	< 0.001
Glasgow coma scale score	15, 1.25	15, 0	< 0.001
Early warning scores at ED arrival			
qSOFA	1.36 ± 0.56	1.08 ± 0.40	< 0.001
MEWS	4.14 ± 1.79	3.19 ± 1.53	< 0.001
NEWS	8.09 ± 2.64	5.75 ± 2.71	< 0.001
REMS	9.80 ± 3.58	6.10 ± 3.48	< 0.001
Outcome			
Mechanical ventilation	109 (42.9)	46 (6.4)	< 0.001
ICU admission	94 (37.0)	91 (12.6)	< 0.001
Length of hospital stay (days)	9.5, 12	10, 8	0.424

Note: data presented as n (%), mean ± SD or median, interquartile range

Abbreviations: CKD, chronic kidney disease; ESRD, end-stage renal disease; ED, emergency department; qSOFA, quick Sequential Organ Failure Assessment; MEWS, Modified Early Warning Score; NEWS, National Early Warning Score; REMS, Rapid Emergency Medicine Score; ICU, intensive care unit

### Scoring systems

**Fig. 1:**
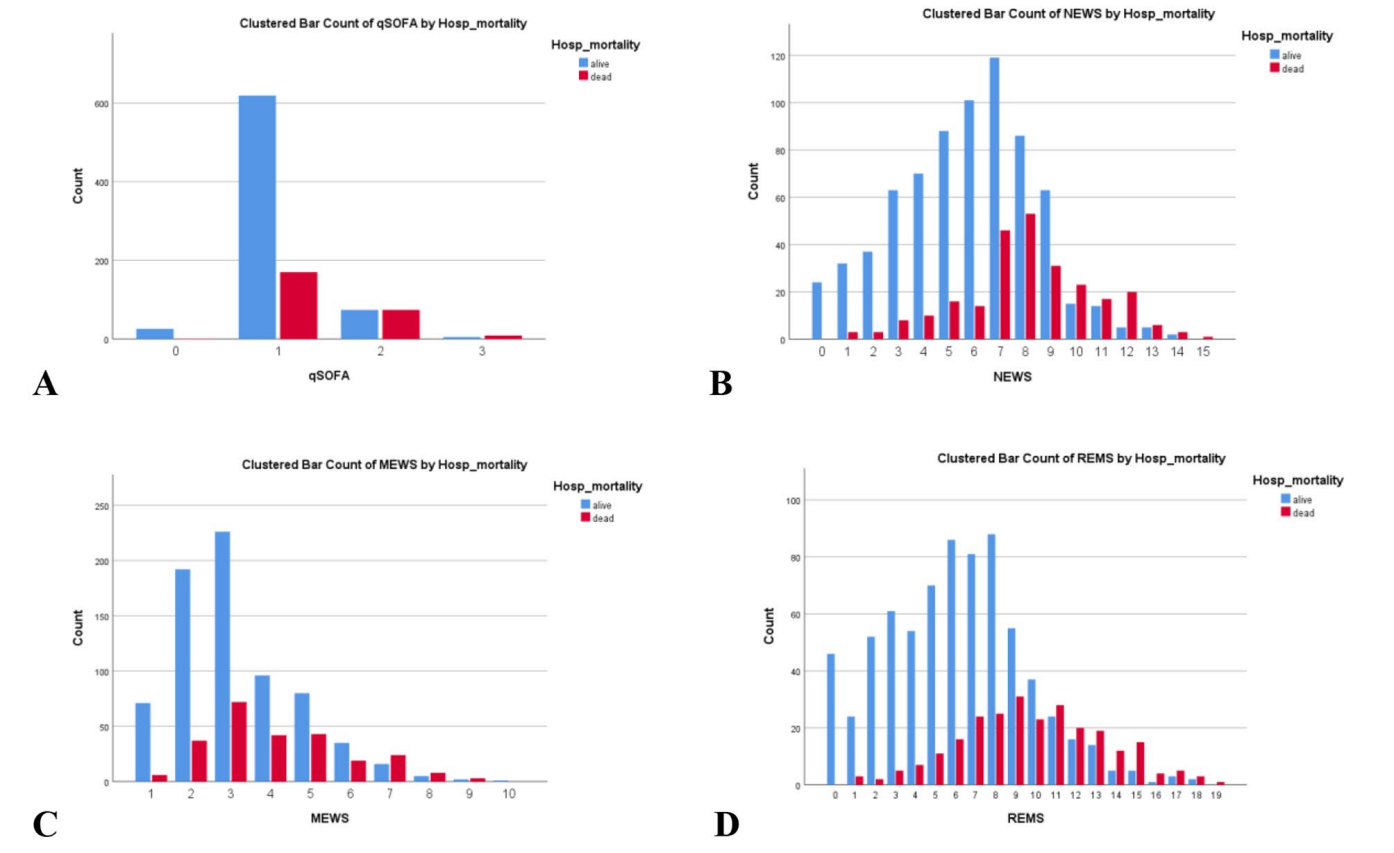
Distribution of early warning scores and in-hospital mortality stratified by each early warning score in emergency patients with COVID-19. (**A**) qSOFA score. (**B**) NEWS score. (**C**) MEWS score. (**D**) REMS score Abbreviations: qSOFA, quick Sequential Organ Failure Assessment; MEWS, Modified Early Warning Score; NEWS, National Early Warning Score; REMS, Rapid Emergency Medicine Score

No patients had missing EWS values. All mean EWS values were significantly higher in those who died at hospital discharge (Table 1). Distributions of scores amongst the cohort are shown in Fig. 1. For all EWSs, a higher proportion of patients with higher scores had met the primary outcome, implying strong associations between EWS values and in-hospital mortality (Fig. 1). However, such an association was not as clear and strong with the secondary outcome of mechanical ventilation (Figure S1).

### Score performance

**Fig. 2:**
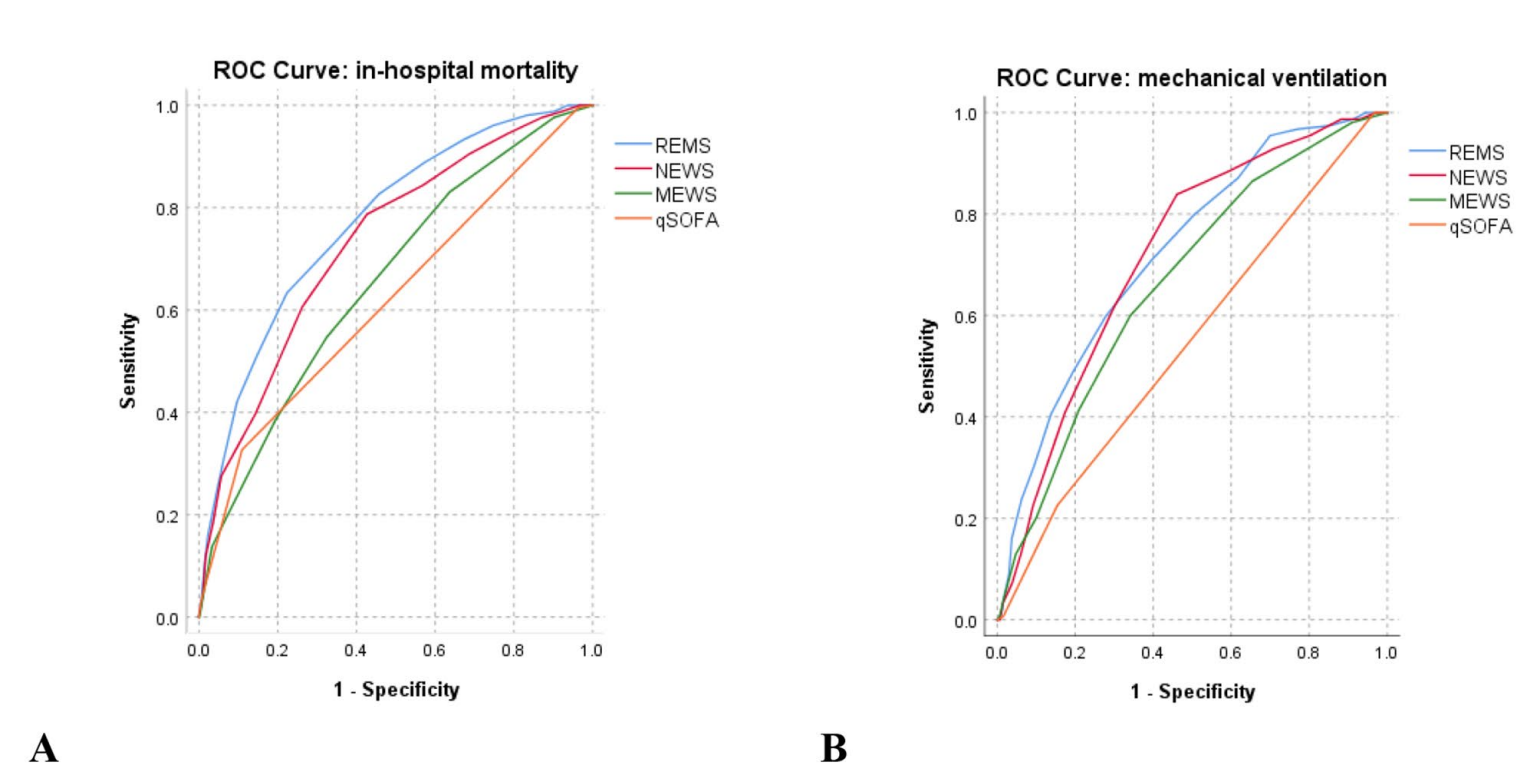
Receiver operator characteristic curves for early warning scores for in-hospital mortality and mechanical ventilation in emergency patients with COVID-19 (**A**) In-hospital mortality. (**B**) Mechanical ventilation. Abbreviations: qSOFA, quick Sequential Organ Failure Assessment; MEWS, Modified Early Warning Score; NEWS, National Early Warning Score; REMS, Rapid Emergency Medicine Score

Based on Nagelkerke’s R square, REMS had the best overall performance, followed by NEWS, qSOFA, and MEWS (Table 2). The discrimination capacity for in-hospital mortality was highest for REMS (AUROC 0.771; 95%CI 0.738, 0.804), followed by NEWS (AUROC 0.732; 95%CI 0.697, 0.767), MEWS (AUROC 0.657; 95%CI 0.619, 0.694), and qSOFA (AUROC 0.620; 95%CI 0.589, 0.651) (Table 2; Fig. 2). REMS, NEWS, and qSOFA had better discrimination based on AUROC for in-hospital mortality than for mechanical ventilation, while MEWS had slightly higher AUROC for mechanical ventilation than for mortality (Table 2). Nonetheless, the trend of results of AUROCs was similar between the primary and secondary outcomes, with REMS having the best discrimination capacity among all EWSs. In pairwise comparisons between EWSs, REMS had significantly better discrimination than all other EWSs for in-hospital mortality (Table 3). It could also yield significantly better discriminating capacity than other EWSs for mechanical ventilation, although its AUROC was not significantly higher than that of NEWS (Table 3). In the subgroup excluding patients with DNR status (n = 750), all EWSs had slightly lower AUROCs compared to those of the whole cohort, with REMS having significantly better discrimination than all other EWSs except for NEWS for both outcomes (Table S2 and S3). Nevertheless, REMS had the best overall performance according to Nagelkerke’s R square (Table S2). Moreover, in the subgroup analysis by study center, REMS had the highest discrimination capacity in all centers for in-hospital mortality; however, it did not outperform NEWS and MEWS for mechanical ventilation in some study centers (Table S4).

**Table 2:** Early warning score performance and clinical utility for in-hospital mortality and mechanical ventilation in emergency patients with COVID-19

	Discrimination	Calibration	Overall performance	Clinical utility						
Score	AUROC (95%CI)	Hosmer-Lemeshow Test	Nagelkerke’s R-Square (%)	Score category	Sensitivity (95%CI)	Specificity (95%CI)	PPV (95%CI)	NPV (95%CI)	LR+ (95%CI)	LR- (95%CI)
**In-hospital mortality**										
qSOFA	0.620 (0.589, 0.651)	0.457	9.3	qSOFA ≥ 2	32.7 (26.9–38.8)	89.1 (86.6–91.3)	51.2 (43.3–59.2)	79.0 (76.1–81.8)	3.0 (2.3–3.9)	0.8 (0.7–0.8)
MEWS	0.657 (0.619, 0.694)	0.559	8.8	MEWS ≥ 4	54.7 (48.4–61.0)	67.5 (64.0-70.9)	37.2 (32.3–42.3)	81.0 (77.6–84.0)	1.7 (1.5-2.0)	0.7 (0.6–0.8)
NEWS	0.732 (0.697, 0.767)	0.156	18.7	NEWS ≥ 7	78.7 (73.2–83.6)	57.3 (53.6–61.0)	39.3 (35.0-43.7)	88.5 (85.2–91.2)	1.8 (1.7–2.1)	0.4 (0.3–0.5)
REMS	0.771 (0.738, 0.804)	0.943	25.0	REMS ≥ 9	63.4 (57.1–69.3)	77.6 (74.4–80.6)	49.8 (44.3–55.4)	85.8 (82.9–88.4)	2.8 (2.4–3.3)	0.5 (0.4–0.6)
**Mechanical ventilation**										
qSOFA	0.547 (0.513, 0.582)	0.074	0.9	qSOFA ≥ 2	22.6 (16.3–30.0)	84.6 (81.9–87.0)	21.6 (15.5–28.7)	85.3 (82.7–87.7)	1.5 (1.1-2.0)	0.9 (0.8-1.0)
MEWS	0.666 (0.623, 0.710)	0.116	6.9	MEWS ≥ 4	60.0 (51.8–67.8)	65.9 (62.5–69.1)	24.9 (20.6–29.6)	89.7 (87.0–92.0)	1.8 (1.5–2.1)	0.6 (0.5–0.7)
NEWS	0.718 (0.678, 0.758)	0.037	11.5	NEWS ≥ 7	83.9 (77.1–89.3)	53.9 (50.5–57.4)	25.5 (21.8–29.6)	94.7 (92.2–96.5)	1.8 (1.7-2.0)	0.3 (0.2–0.4)
REMS	0.724 (0.682, 0.765)	0.322	13.6	REMS ≥ 9	60.0 (51.8–67.8)	72.1 (68.9–75.1)	28.8 (23.9–34.1)	90.5 (88.0-92.7)	2.2 (1.8–2.5)	0.6 (0.5–0.7)

Notes: cut-off values for all early warning scores were chosen by optimal Youden Index.

Abbreviations: AUROC, area under the receiver operator characteristics curve; CI, confidence interval; LR+, positive likelihood ratio; LR-, negative likelihood ratio; NEWS, National Early Warning Score; NPV, negative predictive value; PPV, positive predictive value; qSOFA, quick Sequential Organ Failure Assessment; MEWS, Modified Early Warning Score; NEWS, National Early Warning Score; REMS, Rapid Emergency Medicine Score

**Table 3:** Pairwise comparisons of area under the receiver operator characteristic curve of early warning scores for in-hospital mortality and mechanical ventilation among emergency patients with COVID-19

		**In-hospital mortality**			
		**qSOFA**	**MEWS**	**NEWS**	**REMS**
**Mechanical ventilation**	**qSOFA**		0.063	< 0.001	< 0.001
	**MEWS**	< 0.001		< 0.001	<0.001
	**NEWS**	< 0.001	< 0.001		0.037
	**REMS**	< 0.001	0.012	0.793	

Abbreviations: qSOFA, quick Sequential Organ Failure Assessment; MEWS, Modified Early Warning Score; NEWS, National Early Warning Score; REMS, Rapid Emergency Medicine Score

**Fig. 3:**
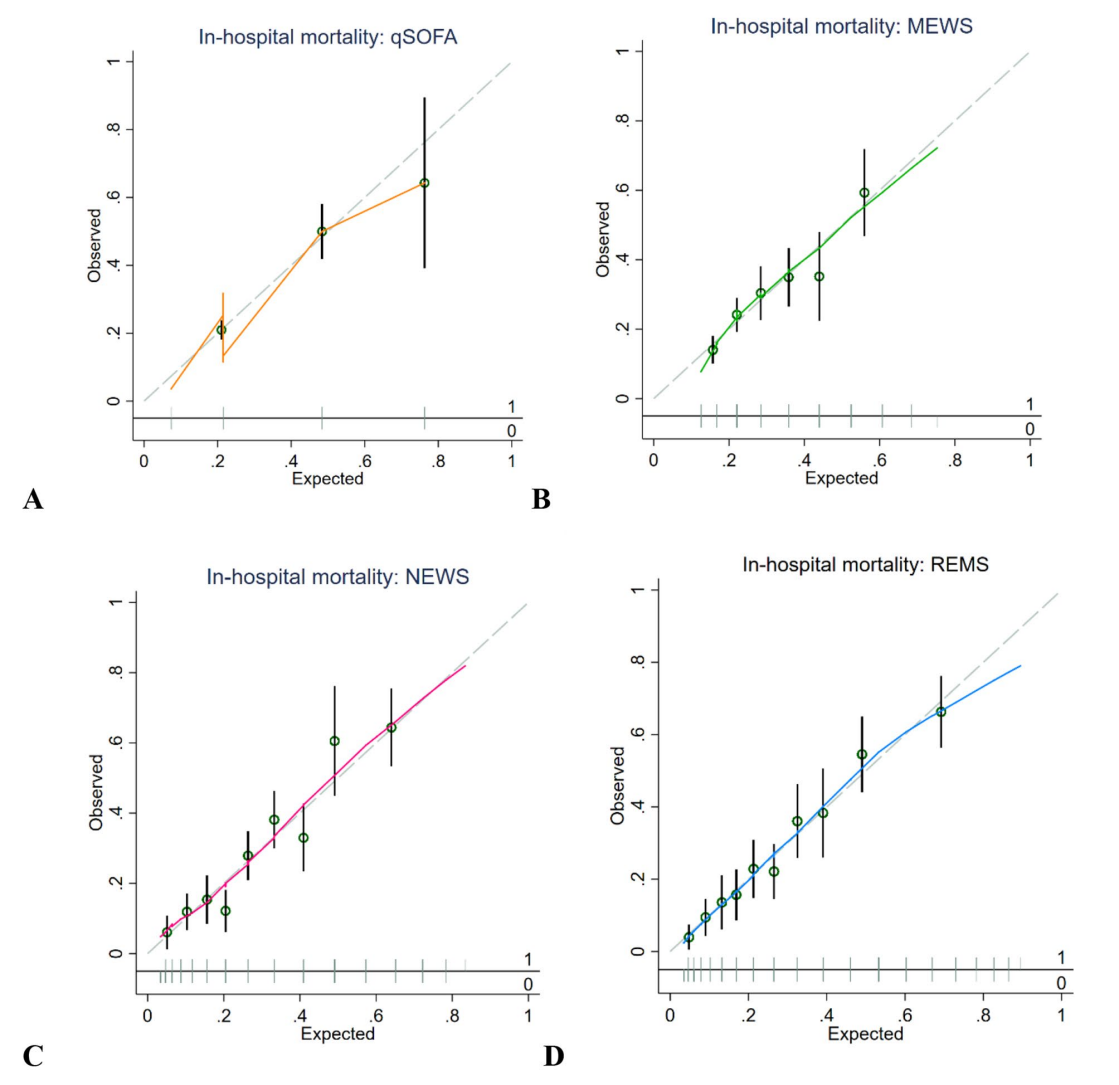
Calibration plots of early warning scores for in-hospital mortality in emergency patients with COVID-19 (**A**) qSOFA score. (**B**) MEWS score. (**C**) NEWS score. (**D**) REMS score. Hollow circles denote groups of predicted risk. Vertical line through hollow circles denote 95% confidence intervals. The distribution of non-events of the outcome (0) and events of the outcome (1) by expected probability are denoted by the rug plot (light grey) along the x axis Abbreviations: qSOFA, quick Sequential Organ Failure Assessment; MEWS, Modified Early Warning Score; NEWS, National Early Warning Score; REMS, Rapid Emergency Medicine Score

Calibration for qSOFA showed mostly an underestimation of the predicted mortality risk (Fig. 3). The other EWSs tended to be well-calibrated except for a slight underestimation of in-hospital mortality risk at high predicted probabilities in REMS (Fig. 3). However, only a few patients had very high REMS scores (Fig. 1). For mechanical ventilation, all EWSs underestimated the risk for the outcome at high predicted probabilities (Figure S2). Calibration based on the Hosmer-Lemeshow tests showed that REMS was the most well-calibrated EWSs among all, with the highest p-value for both outcomes. In the subgroup excluding DNR patients, all EWSs showed an underestimation of predicted mortality and intubation risks at high predicted probabilities except for REMS, which tended to be the most well-calibrated EWS with the least degree of risk underestimation for both outcomes (Figure S3 and S4), concordant with the Hosmer-Lemeshow test results (Table S2).

The results of the clinical usefulness of the EWS scores assessed by sensitivity, specificity, PPV, NPV, LR+, and LR- are shown in Table 2. For both the study outcomes, the optimal cut points based on the Youden index were qSOFA ≥ 2, MEWS ≥ 4, NEWS ≥ 7, and REMS ≥ 9. For mortality, NEWS ≥ 7 had the highest sensitivity but the least specificity. qSOFA ≥ 2 had the highest specificity but lowest sensitivity. REMS ≥ 9 had a balance of sensitivity and specificity that favored sensitivity. All EWSs had higher NPV than PPV, with REMS ≥ 9 also having the most balanced NPV and PPV. REMS ≥ 9 also had the most balanced high LR + and low LR-. For mechanical ventilation, the results of diagnostic accuracy indices were similar to those of the primary outcome (Table 2). In non-DNR subgroup analysis for in-hospital mortality, MEWS ≥ 3 and NEWS ≥ 8 were the optimal cut points. Otherwise, the results of the subgroup for both outcomes were generally comparable to the full cohort, with REMS ≥ 9 having the most balanced diagnostic accuracy indices (Table S2).

64% (n = 509) of all patients had NEWS ≥ 7 but 60.7% (n = 309) did not meet the primary outcome (false positive). Only 16.6% (n = 162) met at least 2 qSOFA criteria (qSOFA ≥ 2), although its proportion of false positives was the lowest (48.8%). MEWS ≥ 7 and REMS ≥ 9 could detect similar proportions of patients (about 33–38%), but REMS ≥ 9 had the lowest false positive rate and the highest absolute risk difference compared to the other EWSs (Table 4). Similarly, REMS ≥ 9 yielded the lowest proportion of false positives in predicting mechanical ventilation (Table 4).

**Table 4:** Classification according to early warning scores

Outcomes	All patients, no (%)	qSOFA, no (%)		MEWS, no (%)		NEWS, no (%)		REMS, no (%)	
		< 2 (n = 816)	≥ 2 (n = 162)	< 4 (n = 604)	≥ 4 (n = 374)	< 7 (n = 469)	≥ 7 (n = 509)	< 9 (n = 655)	≥ 9 (n = 323)
In-hospital death	254 (26.0)	171 (21.0)	83 (51.2)	115 (19.0)	139 (37.2)	54 (11.5)	200 (39.3)	93 (14.2)	161 (49.8)
Mechanical ventilation	155 (15.8)	120 (14.7)	35 (21.6)	62 (10.3)	93 (24.9)	25 (5.3)	130 (25.5)	62 (9.5)	93 (28.8)

Abbreviations: qSOFA, quick Sequential Organ Failure Assessment; MEWS, Modified Early Warning Score; NEWS, National Early Warning Score; REMS, Rapid Emergency Medicine Score

## Discussion

This study was among the first multi-center studies that validated and compared REMS, NEWS, MEWS, and qSOFA in predicting adverse clinical outcomes in patients with COVID-19 in the ED setting. We found that REMS was the EWS with the best performance among all EWSs in predicting in-hospital mortality and mechanical ventilation based on overall performance, discrimination, calibration, and diagnostic accuracy indices.

Identifying COVID-19 patients with high risks of developing adverse outcomes early in the ED is very important. Earlier recognition can not only lead to earlier initiation of effective and appropriate management but also result in appropriate choices of ED disposition. Although many EWSs have been validated for such purpose, [14–21] only a small number of studies with small sample sizes have explored the utility of REMS in prognosticating adverse outcomes for COVID-19 patients in the ED. A single-center study including 137 emergency COVID-19 patients reported that REMS was the EWS with the highest AUROC for in-hospital mortality that was superior to NEWS, MEWS, and qSOFA [22]. Two other single-center studies, each including no more than 350 patients, also showed that REMS had better discrimination than NEWS and/or MEWS in predicting in-hospital mortality [23, 24]. To the best of our knowledge, the present study was the first multi-center study involving a large number of COVID-19 patients in the ED that validated REMS and compared it with other EWSs. The present study yielded similar results to previous studies in terms of discrimination capacity assessed by AUROC, with REMS having the highest discrimination above all the other EWSs for in-hospital mortality. Therefore, the results of the present study may confirm the superior prognosticating ability of REMS over other more commonly-used EWSs. In addition to its superiority in discrimination, we also found that REMS performed the best with overall model performance. Also, the REMS values were well-calibrated and associated with both the study outcomes. Moreover, it could yield the most balanced diagnostic accuracy indices at its optimal cut point. Additionally, the superiority of REMS over other EWSs was consistent in the results of the subgroup analysis excluding DNR patients. The dominance of REMS might have been because of age, which is a component of REMS but not of other EWSs. Older patients might have had higher risks of adverse outcomes secondary to COVID-19 as evidenced by significantly higher mean age among patients discharged dead. In the subgroup analyses by study center, REMS was still the best EWS in terms of discrimination with the highest AUROC in all centers for in-hospital mortality. However, it was not superior to other EWSs for mechanical ventilation. This discordance might have been because of the small number of participants in some study centers and hence small numbers of outcome events. In fact, the subgroup results by study center should be interpreted with caution as most of the subgroups contained < 10 outcome events.

Furthermore, although REMS at its best cut point according to the Youden index could provide the most balanced diagnostic accuracy indices, it is important to note that no EWS has both high sensitivity and specificity. Consequently, the overall prognostic accuracies of these EWSs were not sufficient to be used regardless of clinical signs and symptoms. Clinical correlation should always be considered in conjunction with these EWSs.

Although we could demonstrate the superiority of REMS over other EWSs similar to other previous studies as discussed earlier, it is noticeable that the AUROCs found in the present study were lower than those in previous studies [22–24]. This difference might have been because of the characteristics of the patients, the hospitals, and the healthcare setting specific to Thailand and possibly other middle-income countries. Unlike other high-income countries, we have much more limited healthcare provisions and resources, possibly resulting in a largely higher in-hospital mortality rate than other previous studies conducted in similar patient populations and settings [22, 24, 25]. Besides, some of the all-cause in-hospital mortality encountered by the patients in the present study might not have been caused by COVID-19; thus, this high mortality rate may not truly represent mortality associated with COVID-19. Nonetheless, this issue reflected the importance of the present study as the much higher validation AUROCs in other studies from higher-income countries would have had poor generalizability to our setting. Regardless of such discordance, our results could still emphasize that REMS is a clinically useful EWS for COVID-19 patients in the ED, especially when compared to other more commonly-used EWSs.

There were some limitations to this study. First, despite the study being a multi-center study including EDs of many hospitals with different levels of care, it was conducted in a middle-income country, which may still limit its generalizability. Second, we only included patients who tested positive for COVID-19 within the index admission, so we might have missed some patients who were sent for COVID-19 testing at other testing sites outside of the hospitals due to limited testing capacity and did not revisit the ED before admission. Although the number of patients we might have missed was expected to be very low, including all those eligible patients may better represent the true prognostic value of EWSs. Lastly, we only measured one EWS value upon ED arrival because it was the only time point without any missing data. If repeated measures were available, they might help improve the accuracy of scoring systems. However, such analyses may not have high clinical utility in the ED, especially for COVID-19 situations, where treatment and disposition decisions usually begin at early ED arrival.

## Conclusion

REMS was the EWS with the highest prognostic utility in terms of discrimination, calibration, overall performance, and balanced diagnostic accuracy indices compared to qSOFA, MEWS, and NEWS in predicting in-hospital mortality and mechanical ventilation in COVID-19 patients in the ED. It may be a useful bedside tool to aid in in triage, treatment, and disposition decision-making for emergency COVID-19 patients.

## Electronic supplementary material

Below is the link to the electronic supplementary material.

**Additional file 1: table S1** Components and scores of the qSOFA, NEWS, MEWS, and REMS

**Additional file 2: table S2** Early warning score performance and clinical utility for all-cause in-hospital mortality and mechanical ventilation in emergency patients with COVID-19 without do-not-resuscitate status

**Additional file 3: table S3** Pairwise comparisons of area under the receiver operator characteristic curve of early warning scores for in-hospital mortality and mechanical ventilation among emergency patients with COVID-19 without do-not-resuscitate status

**Additional file 4: table S4** Area under the receiver operator characteristic curves of early warning scores for in-hospital mortality and mechanical ventilation among emergency patients with COVID-19 stratified by study center

**Additional file 5: figure S1** Distribution of early warning scores and mechanical ventilation stratified by each early warning score in emergency patients with COVID-19

**Additional file 6: figure S2** Calibration plots of early warning scores for mechanical ventilation in emergency patients with COVID-19

**Additional file 7: figure S3** Calibration plots of early warning scores for in-hospital mortality in emergency patients with COVID-19 in the subgroup without do-not-resuscitate status

**Additional file 8: figure S4** Calibration plots of early warning scores for mechanical ventilation in emergency patients with COVID-19 in the subgroup without do-not-resuscitate status

## Data Availability

The datasets generated and analysed for this study are not publicly available but are available from the corresponding author upon reasonable request.

## Abbreviations

COVID-19: Coronavirus disease 2019
SARS-CoV-2: Severe acute respiratory syndrome coronavirus 2
ED: Emergency Department
EWS: Early warning score
SIRS: Systemic Inflammatory Response Syndrome criteria
qSOFA: Quick Sequential Organ Failure Assessment
NEWS: National Early Warning Score
MEWS: Modified Early Warning Score
REMS: Rapid Emergency Medicine Score
ICU: Intensive care unit
RT-PCR: Real-time polymerase chain reaction
RR: Respiratory rate
SBP: Systolic blood pressure
PR: Pulse rate
BT: Body temperature
SD: Standard deviation
AUROC: Area under the curve of the receiver operator characteristics curves
CI: Confidence interval
LR+: Positive likelihood ratio
LR-: Negative likelihood ration
NPV: Negative predictive value
PPV: Positive predictive value
DNR: Do-not-resuscitate
