# Supplementary material for: The utility of the Rapid Emergency Medicine Score (REMS) compared with three other early warning scores in predicting in-hospital mortality among COVID-19 patients in the emergency department: a multicenter validation study
Source: BMC Emerg Med. 2023 Apr 26;23:45. doi: 10.1186/s12873-023-00814-w (PMC10132401; doi:10.1186/s12873-023-00814-w)
Supplement: Supplementary file 1 — Additional file 1: table S1 Components and scores of the qSOFA, NEWS, MEWS, and REMS [file 12873_2023_814_MOESM1_ESM.pdf]

Table S1. Components and scores of the qSOFA, NEWS, MEWS, and REMS.

qSOFA (0-3)	NEWS (0-20)	MEWS (0-14)	REMS (0-26)
Altered mentation (1)	HR (0-3)<br>51-90/min (0), 41-50 or 90-110/min (1), 111-130/min (2), ≤40 or >131/min (3)	HR (0-3)<br>51-100/min (0), 40-50 or 101-110/min (1), <40 or 111-129/min (2), ≥130/min (3)	HR (0-4)<br>70-109/min (0), 55-69/min or 110-139/min (2), 40-54/min or 140-179/min (3), ≤ 39/min or ≥ 179/min (4)
RR ≥ 22/min (1)	RR (0-3)<br>12-20/min (0), 9-11/min (1), ≤8 or 21-24/min (2), >25/min (3)	RR (0-3)<br>9-14/min (0), 15-20/min (1), <9 or 21-29/min (2), ≥30/min (3)	RR (0-4)<br>12-24/min (0), 10-11/min or 25-34/min (1), 6-9/min (2), 35-49/min (3), ≤ 5/min or >49/min (4)
SBP≤100 mmHg (1)	Body temperature (0-3)<br>36.1-38°c (0), 35.1-36 or 38.1-39°c (1), ≥39.1°c (2), ≤35°c (3)	Body temperature (0-2)<br>35.0-38.4°c (0), <35°c or ≥38.5°c (2)	Age (0-6)<br>< 45 years (0), 45-54 years (2), 55-64 years (3), 65-74 years (5), > 74 years (6)
	SBP (0-3)<br>111-219 mmHg (0), 101-110 mmHg (1), 91-100 mmHg (2) ≤90 or ≥220 mmHg (3)	SBP (0-3)<br>101-199 mmHg (0), 81-100 mmHg (1), 70-80 or ≥200 mmHg (2), <70 mmHg (3)	MAP (0-4)<br>70-109 mmHg. (0), 50-69 mmHg or 110-129 mmHg. (2), 130-159 mmHg. (3), ≤ 49 mmHg. or >159 mmHg. (4)
	Neurological (0-3)<br>Alert (0), reacting to voice or reacting to pain or unresponsive (3)	Neurological (0-3)<br>Alert (0), reacting to voice (1), reacting to pain (2), unresponsive (3)	Glasgow coma score (0-4)<br>14 or 15 (0), 11-13 (1), 8-10 (2), 5-7 (3), 3 or 4 (4)
	Oxygen saturation (0-3)<br>≥96% (0), 94-95% (1), 92-93% (2), ≤91% (3)		Oxygen saturation (0-4)<br>>89% (0), 86-89% (1), 75-85% (3), <75% (4)
	Oxygen supplement (0-2)<br>No (0), Yes (2)		

Abbreviations: qSOFA, quick sequential organ failure assessment score; NEWS, national early warning score; MEWS; modified early warning score; REMS, rapid emergency medicine score; HR, heart rate; RR, respiratory rate; SBP, systolic blood pressure; MAP, mean arterial pressure; WBC, white blood cell
