# Supplementary material for: The utility of the Rapid Emergency Medicine Score (REMS) compared with three other early warning scores in predicting in-hospital mortality among COVID-19 patients in the emergency department: a multicenter validation study
Source: BMC Emerg Med. 2023 Apr 26;23:45. doi: 10.1186/s12873-023-00814-w (PMC10132401; doi:10.1186/s12873-023-00814-w)
Supplement: Supplementary file 2 — Additional file 2: table S2 Early warning score performance and clinical utility for all-cause in-hospital mortality and mechanical ventilation in emergency patients with COVID-19 without do-not-resuscitate status [file 12873_2023_814_MOESM2_ESM.pdf]

Table S2. Early warning score performance and clinical utility for all-cause in-hospital mortality and mechanical ventilation in emergency patients with COVID-19 without do-not-resuscitate status

	**Discrimination**	**Calibration**	**Overall performance**	**Clinical utility**						
**Score**	**AUROC (95%CI)**	**Hosmer-Lemeshow Test**	**Nagelkerke's R-Square (%)**	**Score category**	**Sensitivity (95%CI)**	**Specificity (95%CI)**	**PPV (95%CI)**	**NPV (95%CI)**	**LR+ (95%CI)**	**LR- (95%CI)**
				**In-hospital mortality**						
qSOFA	0.571 (0.528, 0.613)	0.254	2.8	qSOFA ≥ 2	20.9 (13.1-30.7)	90.4 (87.9-92.6)	23.2 (14.6-33.8)	89.2 (96.6-91.5)	2.2 (1.4-3.5)	0.9 (0.8-1.0)
MEWS	0.636 (0.580, 0.692)	0.231	3.9	MEWS ≥ 3	82.4 (73.0-89.6)	37.9 (34.2-41.8)	15.5 (12.4-19.0)	94.0 (90.4-96.5)	1.3 (1.2-1.5)	0.5 (0.3-0.7)
NEWS	0.701 (0.644, 0.758)	0.039	9.5	NEWS ≥ 8	59.3 (48.5-69.5)	75.3 (71.8-78.5)	24.9 (19.3-31.2)	93.1 (90.6-95.1)	2.4 (1.9-3.0)	0.5 (0.4-0.7)
REMS	0.749 (0.697, 0.802)	0.660	16.0	REMS ≥ 9	57.1 (46.3-67.5)	80.6 (77.3-83.5)	28.9 (22.4-36.1)	93.2 (90.8-95.1)	2.9 (2.3-3.7)	0.5 (0.4-0.7)
				**Mechanical ventilation**						
qSOFA	0.557 (0.520, 0.594)	0.156	1.7	qSOFA ≥ 2	18.2 (11.5-26.7)	90.3 (87.8-92.5)	24.4 (15.6-35.1)	86.5 (83.7-89.0)	1.9 (1.2-3.0)	0.9 (0.8-1.0)
MEWS	0.685 (0.635, 0.736)	0.179	8.6	MEWS ≥ 4	57.3 (47.5-66.7)	70.5 (66.8-74.0)	25.0 (19.8-30.8)	90.6 (87.6-93.0)	1.9 (1.6-2.4)	0.6 (0.5-0.8)
NEWS	0.748 (0.700, 0.795)	0.248	16	NEWS ≥ 7	80.9 (72.3-87.8)	61.4 (57.5-65.2)	26.5 (21.8-31.5)	94.9 (92.4-96.8)	2.1 (1.8-2.4)	0.3 (0.2-0.5)
REMS	0.751 (0.703, 0.799)	0.340	18.9	REMS ≥ 8	65.5 (55.8-74.3)	69.2 (65.5-72.8)	26.8 (21.6-32.5)	92.1 (89.3-94.3)	2.1 (1.8-2.5)	0.5 (0.4-0.7)

Notes: cut-off values for all early warning scores were chosen by optimal Youden Index.

Abbreviations: AUROC, area under the receiver operator characteristics curve; CI, confidence interval; LR+, positive likelihood ratio; LR-, negative likelihood ratio; NEWS, National Early Warning Score; NPV, negative predictive value; PPV, positive predictive value; qSOFA, quick Sequential Organ Failure Assessment; MEWS, Modified Early Warning Score; NEWS, National Early Warning Score; REMS, Rapid Emergency Medicine Score.
