# Supplementary material for: The utility of the Rapid Emergency Medicine Score (REMS) compared with three other early warning scores in predicting in-hospital mortality among COVID-19 patients in the emergency department: a multicenter validation study
Source: BMC Emerg Med. 2023 Apr 26;23:45. doi: 10.1186/s12873-023-00814-w (PMC10132401; doi:10.1186/s12873-023-00814-w)
Supplement: Supplementary file 3 — Additional file 3: table S3 Pairwise comparisons of area under the receiver operator characteristic curve of early warning scores for in-hospital mortality and mechanical ventilation among emergency patients with COVID-19 without do-not-resuscitate status [file 12873_2023_814_MOESM3_ESM.pdf]

Table S3. Pairwise comparisons of area under the receiver operator characteristic curve of early warning scores for in-hospital mortality and mechanical ventilation among emergency patients with COVID-19 without do-not-resuscitate status

		**In-hospital mortality**			
		**qSOFA**	**MEWS**	**NEWS**	**REMS**
**Mechanical ventilation**	**qSOFA**		0.040	<0.001 (0.0001)	<0.001
	**MEWS**	<0.001		0.001 (0.0008)	<0.001 (0.0002)
	**NEWS**	<0.001	<0.001 (0.0003)		0.116
	**REMS**	<0.001	0.017	0.897	

Abbreviations: qSOFA, quick Sequential Organ Failure Assessment; MEWS, Modified Early Warning Score; NEWS, National Early Warning Score; REMS, Rapid Emergency Medicine Score.
