# Supplementary material for: The utility of the Rapid Emergency Medicine Score (REMS) compared with three other early warning scores in predicting in-hospital mortality among COVID-19 patients in the emergency department: a multicenter validation study
Source: BMC Emerg Med. 2023 Apr 26;23:45. doi: 10.1186/s12873-023-00814-w (PMC10132401; doi:10.1186/s12873-023-00814-w)
Supplement: Supplementary file 4 — Additional file 4: table S4 Area under the receiver operator characteristic curves of early warning scores for in-hospital mortality and mechanical ventilation among emergency patients with COVID-19 stratified by study center [file 12873_2023_814_MOESM4_ESM.pdf]

Table S4. Area under the receiver operator characteristic curves of early warning scores for in-hospital mortality and mechanical ventilation among emergency patients with COVID-19 stratified by study center

	**Center 1 Siriraj Hospital (n=613)**	**Center 2 Banphaeo Hospital (n=244)**	**Center 3 Ratchaburi Hospital (n=60)**	**Center 4 Buddhachinaraj Hospital (n=31)**	**Center 5 Prachuap Khiri Khan Hospital (n=30)**
			**In-hospital mortality**		
qSOFA	0.621 (0.582, 0.661)	0.654 (0.579, 0.730)	0.532 (0.469, 0.595)	0.726 (0.563, 0.889)	0.648 (0.455, 0.840)
MEWS	0.635 (0.585, 0.684)	0.670 (0.589, 0.750)	0.752 (0.630, 0.874)	0.674 (0.451, 0.897)	0.855 (0.717, 0.993)
NEWS	0.720 (0.673, 0.767)	0.751 (0.678, 0.825)	0.797 (0.680, 0.913)	0.660 (0.465, 0.854)	0.903 (0.793, 1.0)
REMS	0.764 (0.721, 0.806)	0.790 (0.720, 0.860)	0.895 (0.820, 0.970)	0.829 (0.644, 1.0)	0.821 (0.630, 1.0)
			**Mechanical ventilation**		
qSOFA	0.552 (0.509, 0.595)	0.675 (0.238, 1.0)	0.533 (0.470, 0.597)	0.60 (0.395, 0.804)	0.597 (0.385, 0.809)
MEWS	0.627 (0.572, 0.683)	0.896 (0.710, 1.0)	0.769 (0.649, 0.889)	0.583 (0.293, 0.874)	0.844 (0.684, 1.0)
NEWS	0.697 (0.645, 0.748)	0.936 (0.844, 1.0)	0.808 (0.692, 0.924)	0.543 (0.321, 0.766)	0.830 (0.687, 0.973)
REMS	0.669 (0.614, 0.724)	0.816 (0.487, 1.0)	0.892 (0.816, 0.968)	0.750 (0.462, 1.0)	0.726 (0.482, 0.969)

Abbreviations: qSOFA, quick Sequential Organ Failure Assessment; MEWS, Modified Early Warning Score; NEWS, National Early Warning Score; REMS, Rapid Emergency Medicine Score.
