# Supplementary material for: The utility of the Rapid Emergency Medicine Score (REMS) compared with three other early warning scores in predicting in-hospital mortality among COVID-19 patients in the emergency department: a multicenter validation study
Source: BMC Emerg Med. 2023 Apr 26;23:45. doi: 10.1186/s12873-023-00814-w (PMC10132401; doi:10.1186/s12873-023-00814-w)
Supplement: Supplementary file 5 — Additional file 5: figure S1 Distribution of early warning scores and mechanical ventilation stratified by each early warning score in emergency patients with COVID-19 [file 12873_2023_814_MOESM5_ESM.pdf]

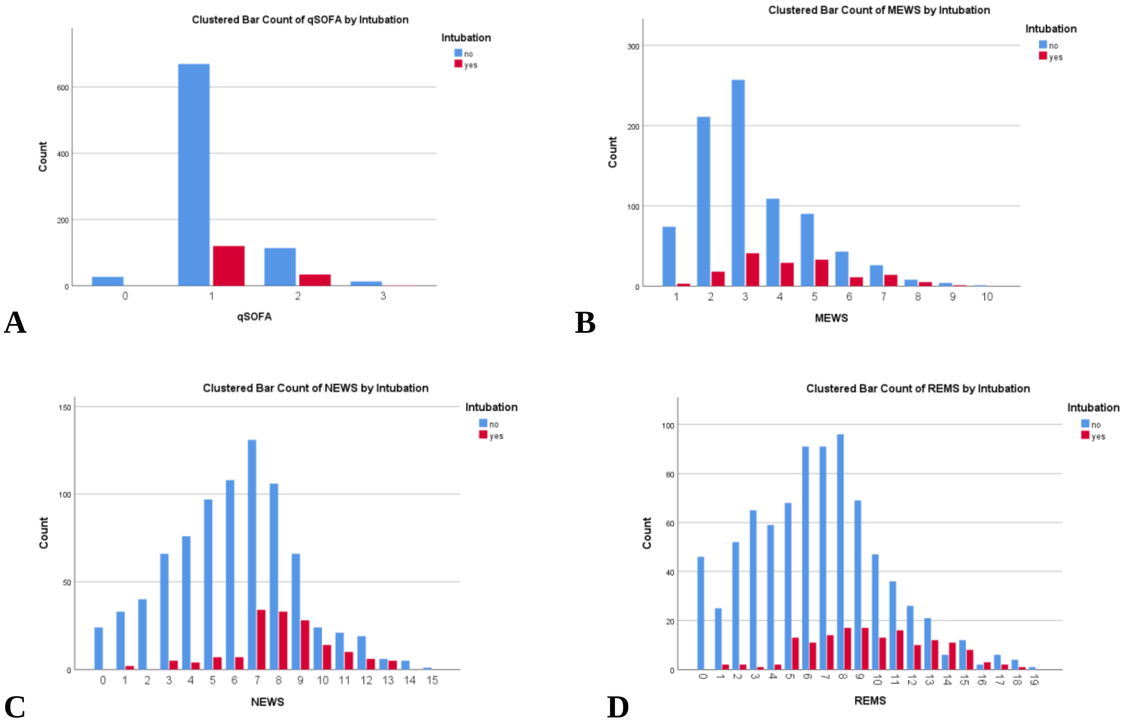

Figure S1. Distribution of early warning scores and mechanical ventilation stratified by each early warning score in emergency patients with COVID-19.

(A) qSOFA score. (B) MEWS score. (C) NEWS score. (D) REMS score.

Abbreviations: qSOFA, quick Sequential Organ Failure Assessment; MEWS, Modified Early Warning Score; NEWS, National Early Warning Score; REMS, Rapid Emergency Medicine Score.
