# Supplementary material for: The utility of the Rapid Emergency Medicine Score (REMS) compared with three other early warning scores in predicting in-hospital mortality among COVID-19 patients in the emergency department: a multicenter validation study
Source: BMC Emerg Med. 2023 Apr 26;23:45. doi: 10.1186/s12873-023-00814-w (PMC10132401; doi:10.1186/s12873-023-00814-w)
Supplement: Supplementary file 7 — Additional file 7: figure S3 Calibration plots of early warning scores for in-hospital mortality in emergency patients with COVID-19 in the subgroup without do-not-resuscitate status [file 12873_2023_814_MOESM7_ESM.pdf]

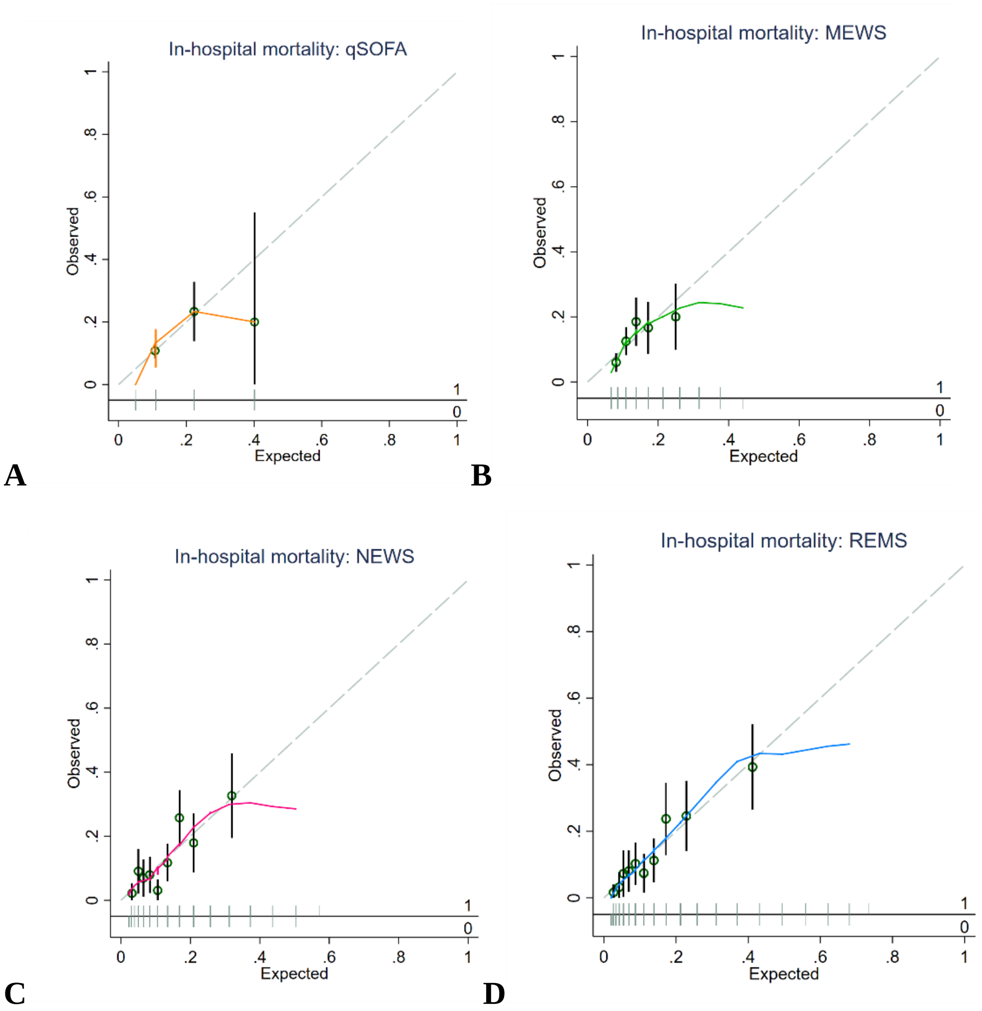

Figure S3. Calibration plots of early warning scores for in-hospital mortality in emergency patients with COVID-19 in the subgroup without do-not-resuscitate status

(A) qSOFA score. (B) MEWS score. (C) NEWS score. (D) REMS score. Hollow circles denote groups of predicted risk. Vertical line through hollow circles denote 95% confidence intervals. The distribution of non-events of the outcome (0) and events of the outcome (1) by expected probability are denoted by the rug plot (light grey) along the x axis.

Abbreviations: qSOFA, quick Sequential Organ Failure Assessment; MEWS, Modified Early Warning Score; NEWS, National Early Warning Score; REMS, Rapid Emergency Medicine Score.
